# The Predictive Potential of the Baseline C-Reactive Protein Levels for the Efficiency of Immune Checkpoint Inhibitors in Cancer Patients: A Systematic Review and Meta-Analysis

**DOI:** 10.3389/fimmu.2022.827788

**Published:** 2022-02-08

**Authors:** Cheng-Long Han, Guang-Xiao Meng, Zi-Niu Ding, Zhao-Ru Dong, Zhi-Qiang Chen, Jian-Guo Hong, Lun-Jie Yan, Hui Liu, Bao-Wen Tian, Long-Shan Yang, Jun-Shuai Xue, Tao Li

**Affiliations:** ^1^ Department of General Surgery, Qilu Hospital, Shandong University, Jinan, China; ^2^ Department of Hepatobiliary Surgery, The Second Hospital of Shandong University, Jinan, China

**Keywords:** predictive potential, C-reactive protein, prognosis, immune checkpoint inhibitors, cancer, meta-analysis

## Abstract

**Background:**

The relationship between baseline C-reactive protein (CRP) level and the prognosis of cancer patients receiving immune checkpoint inhibitor (ICI) treatment remains controversial. The aim of this meta-analysis was to clarify whether baseline CRP level can serve as a biomarker to predict the efficiency of ICI therapy.

**Methods:**

All associated articles published in the Cochrane Library, EMBASE, and PubMed databases from the inception of the database to December 30, 2021, were retrieved. Progression-free survival (PFS) and overall survival (OS) outcomes were meta-analyzed using the random-effects model and adjusted using the trim-and-fill method because of publication bias.

**Results:**

Thirty-three studies (6,124 patients) conducted between 2013 and 2021 were identified. The pooled outcomes implied that high baseline CRP level patients had significantly worse OS (adjusted pooled value for univariate and multivariate analysis outcomes: HR = 1.48, 95% CI = 1.41–1.56; HR = 1.46, 95% CI = 1.34–1.59) and PFS (adjusted pooled value for univariate and multivariate analysis outcomes: HR = 1.29, 95% CI = 1.15–1.45; HR = 1.20, 95% CI = 1.02–1.40) than low baseline CRP level patients, irrespective of cancer or ICI type. Further analysis indicated that 1 mg/dl was appropriate as a cutoff value for determining the low or high level of baseline CRP to predict the OS or PFS of cancer patients receiving ICI treatment (univariate analysis: HR = 1.56, 95% CI = 1.24–1.97, *P* = 0.909; multivariate analysis: HR = 1.58, 95% CI = 1.23–2.03, *P* = 0.521).

**Conclusions:**

High baseline CRP level (>1 mg/dl) may be an indicator for worse OS and PFS of cancer patients treated with ICIs. More high-quality prospective studies are warranted to assess the predictive value of CRP for ICI treatment.

## Highlights

- Our study provides a comprehensive review and meta-analysis and indicates that high baseline CRP level (>1 mg/dl) may be a good predictor for recurrence and worse survival of cancer patients who received ICI treatment.

## Introduction

Recently, immune checkpoint inhibitors (ICIs) have been more and more commonly applied in clinical use for cancer patients. ICIs mainly contain monoclonal antibodies against programmed cell death 1 (PD-1), its primary ligand (PD-L1), and cytotoxic T lymphocyte-associated protein 4 (CTLA-4) (1, 2). ICIs operate predominantly by deregulating the function of T cells and other immune cells and have shown remarkable effectiveness in the treatment of multiple solid malignancies (3–8). However, some tumors showed intrinsic resistance to ICIs which has gravely restricted the efficiency of ICIs (9). The exact resistance mechanism remains to be determined and may comprise multiple drivers in the light of contemporary findings (2). Therefore, in the area of ICI treatment, it is critical to identify predictors which can specifically anticipate the curative benefits of ICIs. With these predictors, we can guarantee the application of ICIs and presume the concrete resistance mechanisms to furnish orientation toward the subsequent resolution of the resistance mechanism.

Persistent inflammation is increasingly recognized to cause or contribute to immunosuppression (10, 11), which will impair the effect of ICIs or even lead to resistance in cancer patients. Thus, inflammatory biomarkers, including C-reactive protein (CRP), may be reliable prognostic biomarkers for cancer patients receiving ICI treatment. CRP was initially identified in 1930 as a serum protein, which is synthesized in the liver and vigorously responds to the capsular (“C”) polysaccharide of pneumococcus (12). There have previously been plenty of meta-analyses corroborating the correlation between elevated baseline CRP level and poor outcomes of patients with various cancers (13–16). In contrast, there is no confirmatory evidence-based medical research on whether the predictive effect of CRP is applicable to cancer patients treated with ICIs. Numerous studies have demonstrated the predictive value of CRP on ICI treatment (17), yet some studies also exist which argue that CRP lacks predictive power; thus, the existence and definite magnitude of the predictive power of CRP on the prognosis of cancer patients treated with ICIs are controversial and remain to be determined.

Herein, according to the 33 implemented studies, a meta-analysis was conducted. The corrected consolidated hazard ratio (HR) with 95% confidence interval (95% CI) was used to verify the correlations between pretreatment CRP level and overall survival (OS) and progression-free survival (PFS) of cancer patients to assess whether CRP could serve as a predictive biomarker for cancer patients receiving ICI treatment.

## Methods

This study was undertaken according to the Preferred Reporting Items for Systematic Reviews and Meta-Analyses (PRISMA) guidelines (18), and the selection criteria were established following the PICOS model (population, intervention, comparison, outcome, and study design).

### Search Strategies and Selection Criteria

Suitable studies were searched from the Cochrane Library literature, PubMed, and EMBASE databases from the inception of the database until December 30, 2021, and the language was restricted to English. Overall, 33 studies conducted between 2013 and 2021 were obtained. Carcinoma, neoplasm, malignancy, cancer, C-reactive protein, CRP, immune checkpoint inhibitor, ICIs, avelumab, durvalumab, tremelimumab, pembrolizumab, camrelizumab, ipilimumab, tislelizumab, SHR-1210, toripalimab, penpulimab, nivolumab, atezolizumab, PD-1, PD-L1, and CTLA-4 were employed as the literature search keywords. The detailed search strategy and retrieval methods are presented in **Supplementary Table 1**.

The selection criteria were as follows: 1) patients were diagnosed with cancer and treated with ICIs; 2) correlations between CRP and prognostic outcomes, such as OS or PFS, were assessed in the form of the HR with 95% CI; 3) published in English; and 4) no duplicate publication of data. For republished studies, only the studies with the most comprehensive data were collected.

### Data Extraction and Quality Assessment

Three independent reviewers (C-LH, L-JY, and HL) evaluated the availability of each study, and disagreements were discussed and addressed with B-WT. The following data were retrieved for each study: study ID (last name of the first author plus publication year), country, study period, data collection, ICIs, cancer type, sample size, outcome, cutoff value, and HR and 95% CI for OS and PFS derived from univariate analysis or multivariate analysis.

We appraised study quality using the Newcastle-Ottawa Scale criteria (19). Studies are divided into high- (scores greater than 7), medium- (scores within 5 to 7), and low-quality (scores less than 5) studies.

### Statistical Analysis

Data analysis was conducted by Stata 12.0 (Stata Corp LP, College Station, TX). *P <*0.05 was regarded as statistically significant, and the random-effects model was used. Gross variation was categorized by dissimilarity (I2); amounts greater than 25%, 50%, to 75% were considered minor, moderate, to large. The OS and PFS were compared between high baseline CPR group and low baseline CPR group through pooled HR and 95% CI. If removal of a study outcome in the sensitivity analysis results in a significant bias of the pooled HR and 95% CI, the outcome will be eliminated. A funnel plot is a visual tool for testing publication bias wherein the plot should resemble a symmetrical inverted funnel when lacking publication bias (20); otherwise, it is asymmetrical. We identified significant publication bias through funnel plots and ultimately obtained the adjusted pooled HR and 95% CI by the trim-and-fill method to eliminate publication bias. Subgroup analyses were performed by cancer type, sample size, country, and ICI type to determine the potential sources of heterogeneity.

## Results

### Literature Selection and Study Characteristics

**Figure 1:**
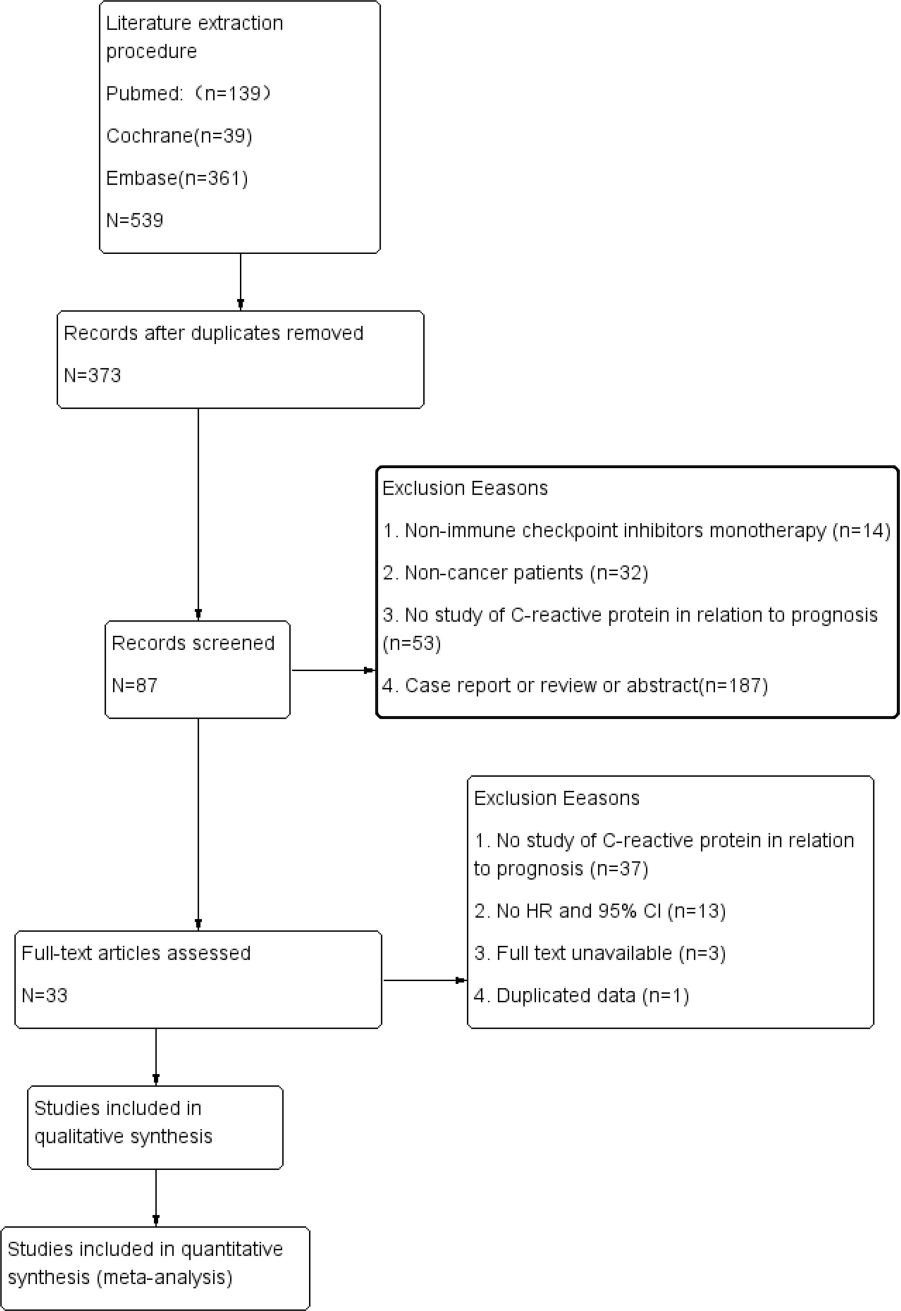
Flowchart of this meta–analysis.

The extraction procedure of relevant literature is shown in **Figure 1**. Initially, 539 records were selected, leaving 373 studies after eliminating duplicates. Following initial screening by title and abstract, 286 papers were excluded. Then, 54 studies were removed from the full-text review due to lack of expected outcomes, duplicated data, or unavailability of full text, resulting in a pool of 33 suitable studies published between 2013 and 2021 (21–53). All studies covered a range of 11 countries, 9 types of cancer, 6 types of ICIs, and 6,124 individuals, as detailed in **Table 1**. There were only 2 studies with NOS scores of 4, while the rest had NOS scores between 5 and 7 (**Supplementary Table 2**).

**Table 1:** Characteristics of the included studies.

Study ID	Study period	Data collection	Country	Cancer type	ICIs	Sample size	Outcome	NOS
Yamamoto–2021	2015–2019	Retrospective	Japan	UC	Pembro	121	OS	7
Tamura–2020	2018–2019	Retrospective	Japan	UC	Pembro	41	OS	5
Wang–2019	2016–2017	Retrospective	China	ESCC	Camre	43	OS	6
Aamdal–2021	2014–2015	Prospective	Norway	Melanoma	Ipi	151	OS	6
Arends–2021	NR	Prospective	UK	HNSCC	Durva	158	OS	6
Fujiwara–2021	2018–2020	Retrospective	Japan	UC	Pembro	74	OS	7
Heppt–2017	2016	Retrospective	Germany	Melanoma	Pembro, Nivo, Ipi	95	OS	7
Hopkins–2020	NR	NR	Australia	NSCLC	Atezo	751	OS, PFS	6
Laino–2020^a^	NR	NR	USA	Melanoma	Ipi, Nivo	1,295	OS	6
Oya–2017	NR	Retrospective	Japan	NSCLC	Nivo	124	PFS	5
Roussel–2021	NR	Retrospective	Belgium	RCC	Nivo	113	OS, PFS	7
Sato–2021	2017–2019	Retrospective	Japan	GC	Nivo	278	OS	4
Wilgenhof–2013	2010–2011	NR	Belgium	Melanoma	Ipi	50	OS	5
Yasuoka–2019	2018	Retrospective	Japan	UC	Pembro	40	OS	5
Awada–2021	2014–2019	Prospective	Belgium	Melanoma	Pembro	183	OS, PFS	6
Chasseuil–2018	2013–2016	Retrospective	France	Melanoma	Nivo	87	OS, PFS	5
Nakamura–2016	2014–2016	Retrospective	Japan	Melanoma	Nivo	98	OS, PFS	7
Niwa–2020	NR	Retrospective	Japan	SGC	Nivo	24	OS, PFS	7
Shoji–2019	2015–2019	NR	Japan	NSCLC	Nivo, Pembro, Atezo	102	OS, PFS	7
Tanizaki–2018	2015–2016	NR	Japan	NSCLC	Nivo	134	OS, PFS	7
Riedl–2020^b^	NR	Retrospective	Austria	NSCLC	ICIs	191	OS, PFS	7
Carbone–2019	NR	Retrospective	Italy	NSCLC	Nivo	72	OS	4
Adachi–2020	2016–2018	Retrospective	Japan	NSCLC	Nivo	296	PFS	6
Inomata–2020	NR	Retrospective	Japan	NSCLC	Nivo, Pembro	36	PFS	5
Noguchi–2020	NR	NR	Japan	RCC	Nivo	64	PFS	7
Shirotake–2019	2016–2018	Retrospective	Japan	RCC	Nivo	54	PFS	7
Suzuki–2020	2016–2019	Retrospective	Japan	RCC	Nivo	65	OS, PFS	7
Takeyasu–2021	2017–2020	Retrospective	Japan	NSCLC	Pembro	145	PFS	6
Tsutsumida–2019	2017–2018	Retrospective	Japan	Melanoma	Nivo then Ipi	55	PFS	5
Ishihara–2019	2013–2019	Retrospective	Japan	RCC	Nivo	58	OS, PFS	6
Katayama–2019	2015–2018	Retrospective	Japan	NSCLC	ICIs	40	OS	5
Scheiner–2021	2015–2020	Retrospective	Austria, Germany	HCC	ICIs	190	OS	7
Abuhelwa–2021^c^	NR	Retrospective	Austria	UC	Atezo	896	OS	6

a This study contains six sets of appropriate outcomes.

b This study contains two sets of appropriate outcomes.

c This study contains two sets of appropriate outcomes.

### Relationship Between Baseline CRP and OS in Cancer Patients Treated With ICIs

The cutoff value of high and low baseline CRP level groups was ascertained according to the study-specific cutoff values. The cutoff values of CRP in all 33 studies are listed in **Supplementary Table 3**, and the cutoff value of most of the studies was not higher than 1 mg/dl. Among these 33 studies, 3 studies did not report the cutoff value of CRP, 11 studies used 1 mg/dl as the cutoff value, 4 studies used median CRP level as the cutoff value, while the other studies used cutoff values according to the study-specific consideration. In terms of median CRP level, 10 studies with 2,501 patients had median CRP level <1 mg/dl, and 8 studies with 1,541 patients had median CRP level >1 mg/dl (**Supplementary Table 4**).

**Figure 2:**
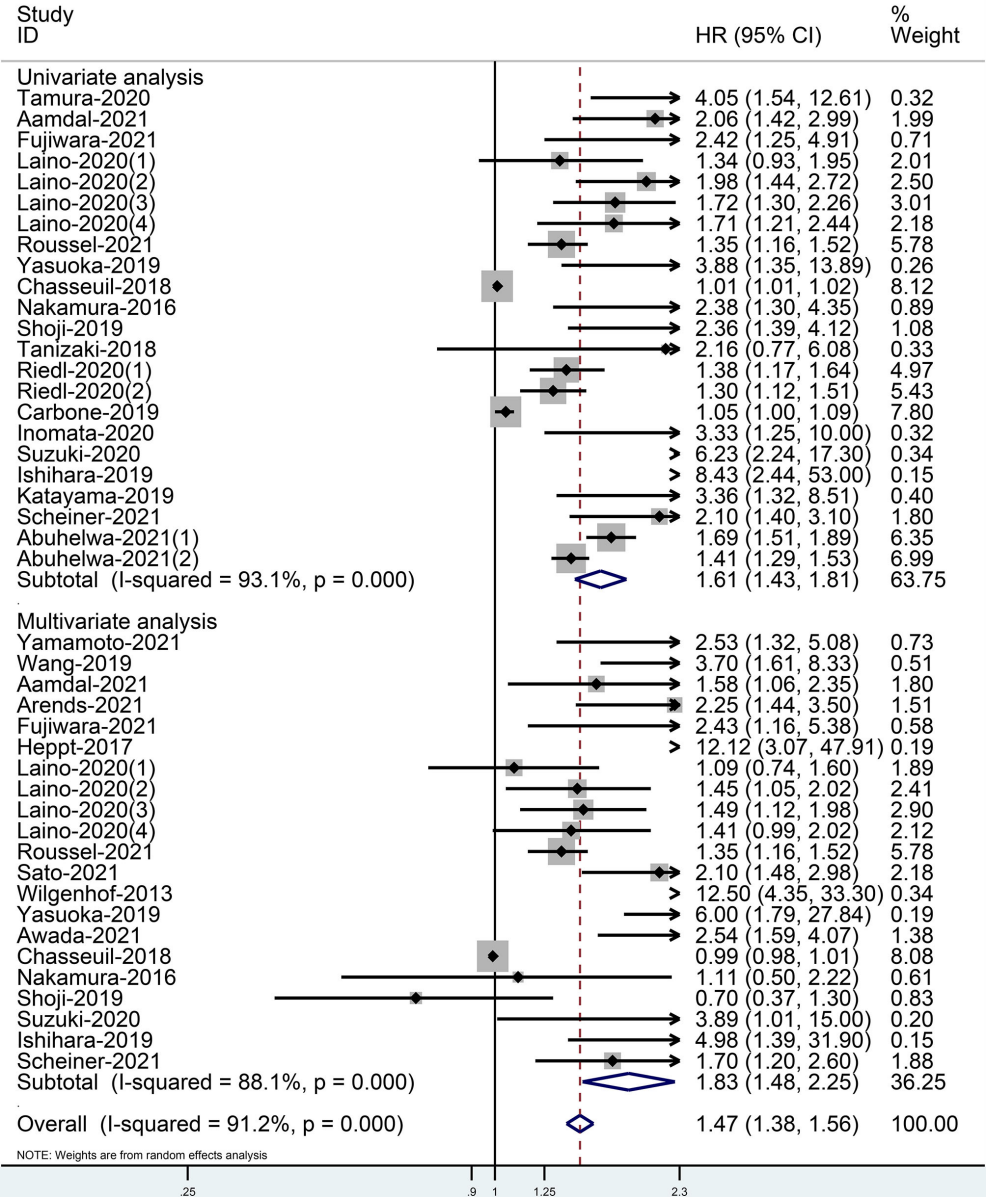
Forest plot of pooled HR and 95% CI about the relationship of baseline CRP levels and OS categorized by univariate and multivariate analysis outcomes in cancer patients treated with ICIs (squares = HR, horizontal lines = 95% CI, rhombus = summarized HR and its 95% CI).

**Figure 3:**
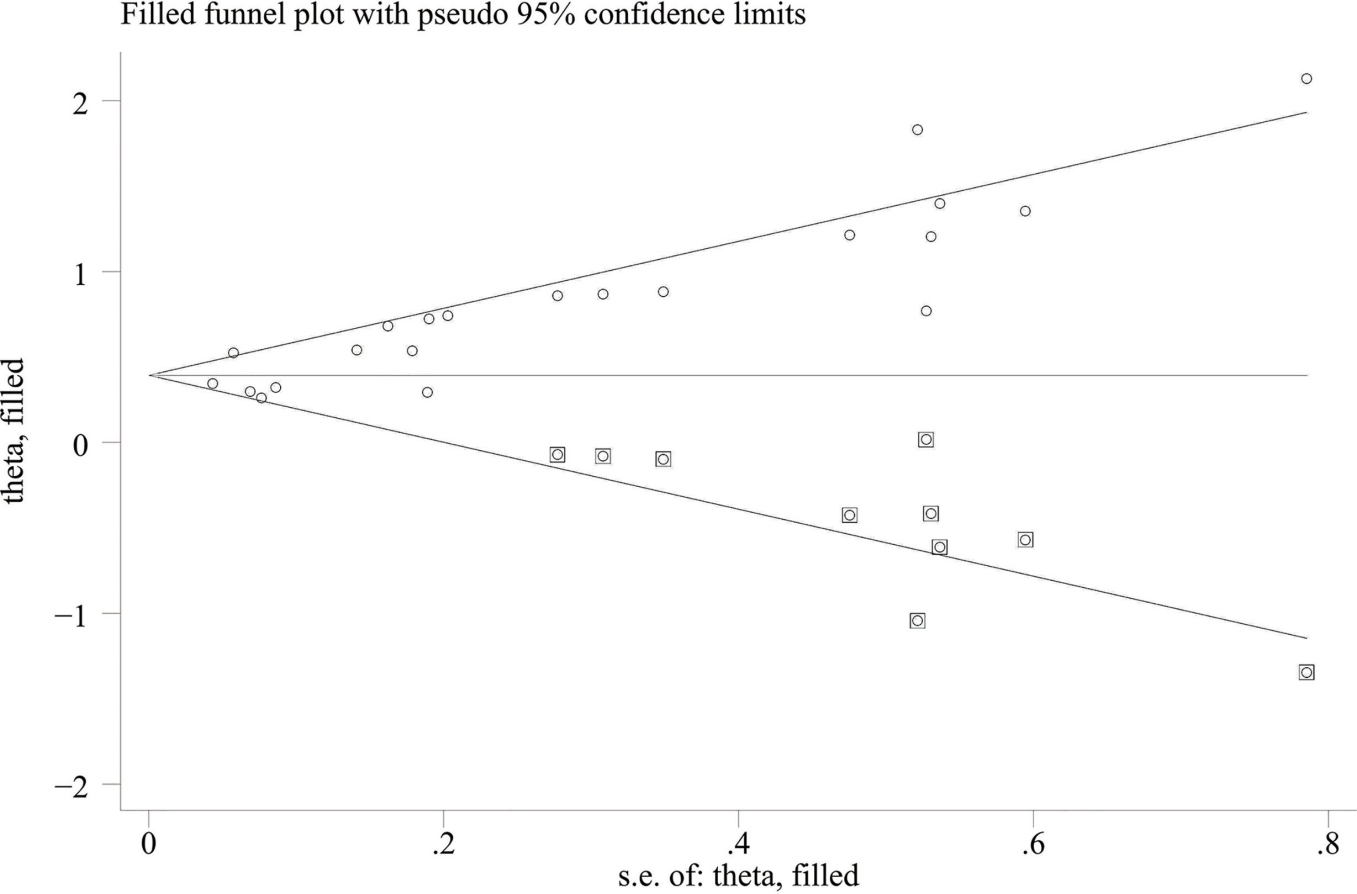
Trimming chart of univariate analysis outcomes of baseline CRP levels and OS [round dots = the observed studies, square dots = the missing studies imputed by the trim–and–fill method, solid lines that create a triangular area indicate the 95% CI (under the fixed–effect model), and the horizontal solid line represents the overall effect size.

**Figure 4:**
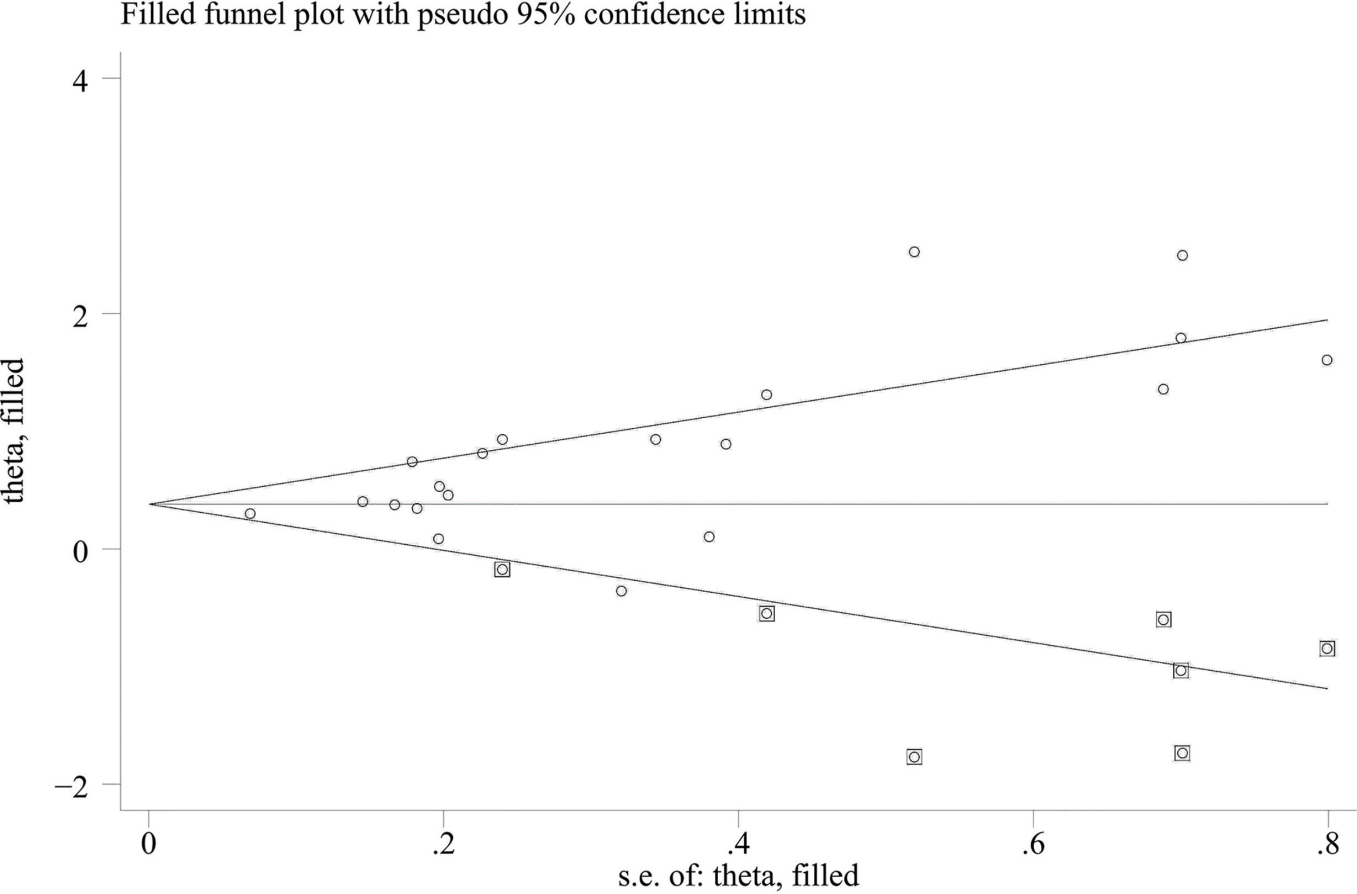
Trimming chart of multivariate analysis outcomes of baseline CRP levels and OS.

When comparing the high baseline CRP level group with the low baseline CRP level group, the pooled outcomes of univariate and multivariate analyses of worse OS were 1.61 (95% CI = 1.43–1.81) and 1.83 (95% CI = 1.48–2.25), respectively (**Figure 2**). After sensitivity analysis, we identified that the outcomes of the studies of Chasseuil et al. (35) and Carbone et al. (41) caused significant bias in the pooled HR and 95% CI, and therefore had to be discarded. The results before and after removing the biased outcomes can be observed in **Supplementary Figures 1**-**5**. The funnel plots indicated that there existed obvious publication bias in the available data, and many unpublished negative results were likely missing (**Supplementary Figures 6, 7**), so we finally utilized the trim-and-fill method to obtain the corrected pooled values for HR and 95% CI of OS. The adjusted pooled value for univariate analysis outcomes is 1.48 (95% CI = 1.41–1.56), and the corrected pooled value for multivariate analysis outcomes is 1.46 (95% CI = 1.34–1.59) (**Figures 3**, **4**). Further analysis indicated that 1 mg/dl was appropriate as the cutoff value for determining low or high level of the baseline CRP to predict the OS of cancer patients receiving ICI treatment (univariate analysis group: HR = 2.34, 95% CI = 1.77–3.08, *P* = 0.355; multivariate analysis group: HR = 1.92, 95% CI = 1.43–2.56, *P* = 0.256) (**Supplementary Figure 8**).

### Relationship Between Baseline CRP and PFS in Cancer Patients Treated With ICIs

**Figure 5:**
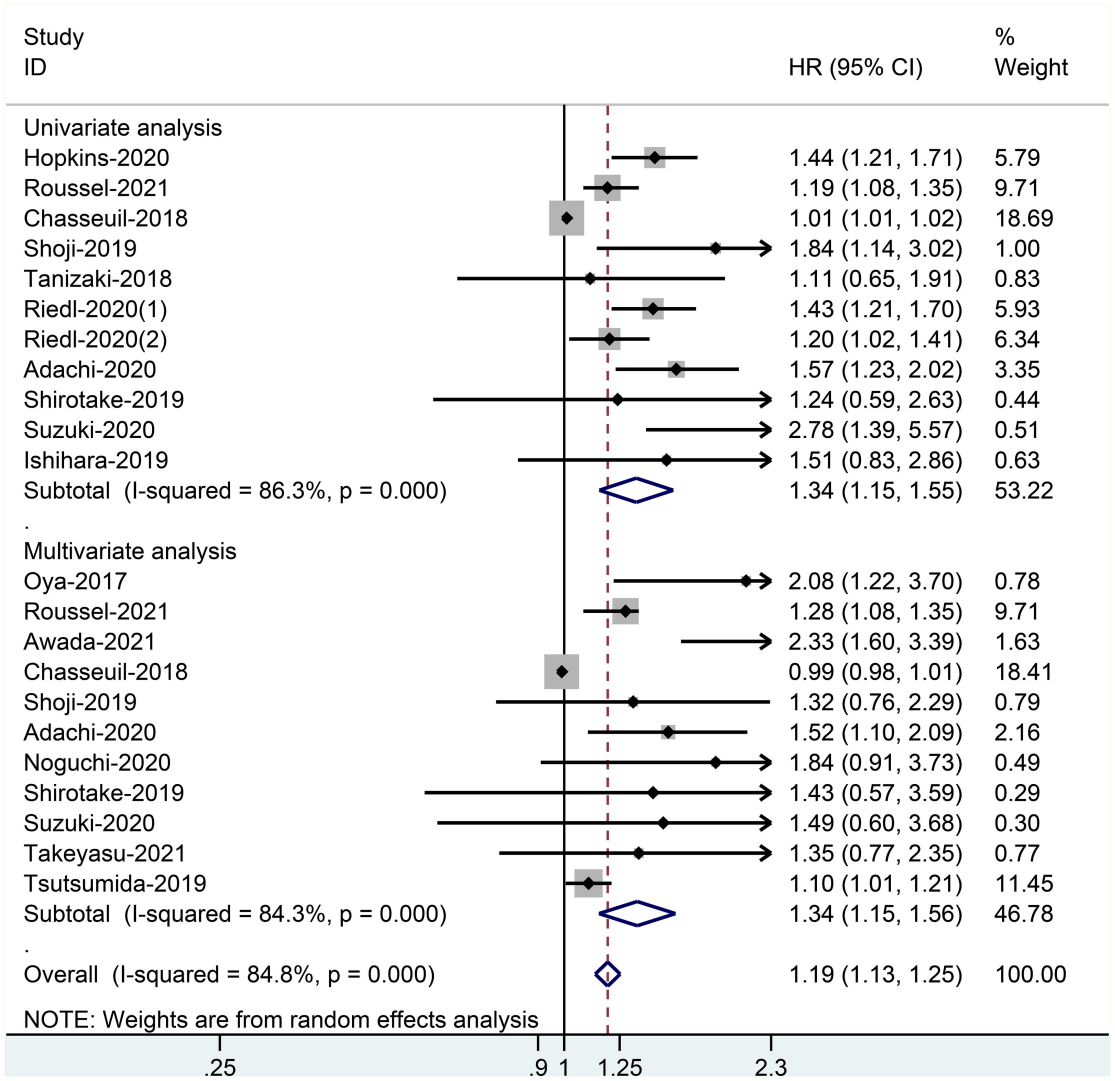
Forest plot of pooled HR and 95% CI about the relationship of baseline CRP levels and PFS categorized by univariate and multivariate analysis outcomes in cancer patients treated with ICIs.

**Figure 6:**
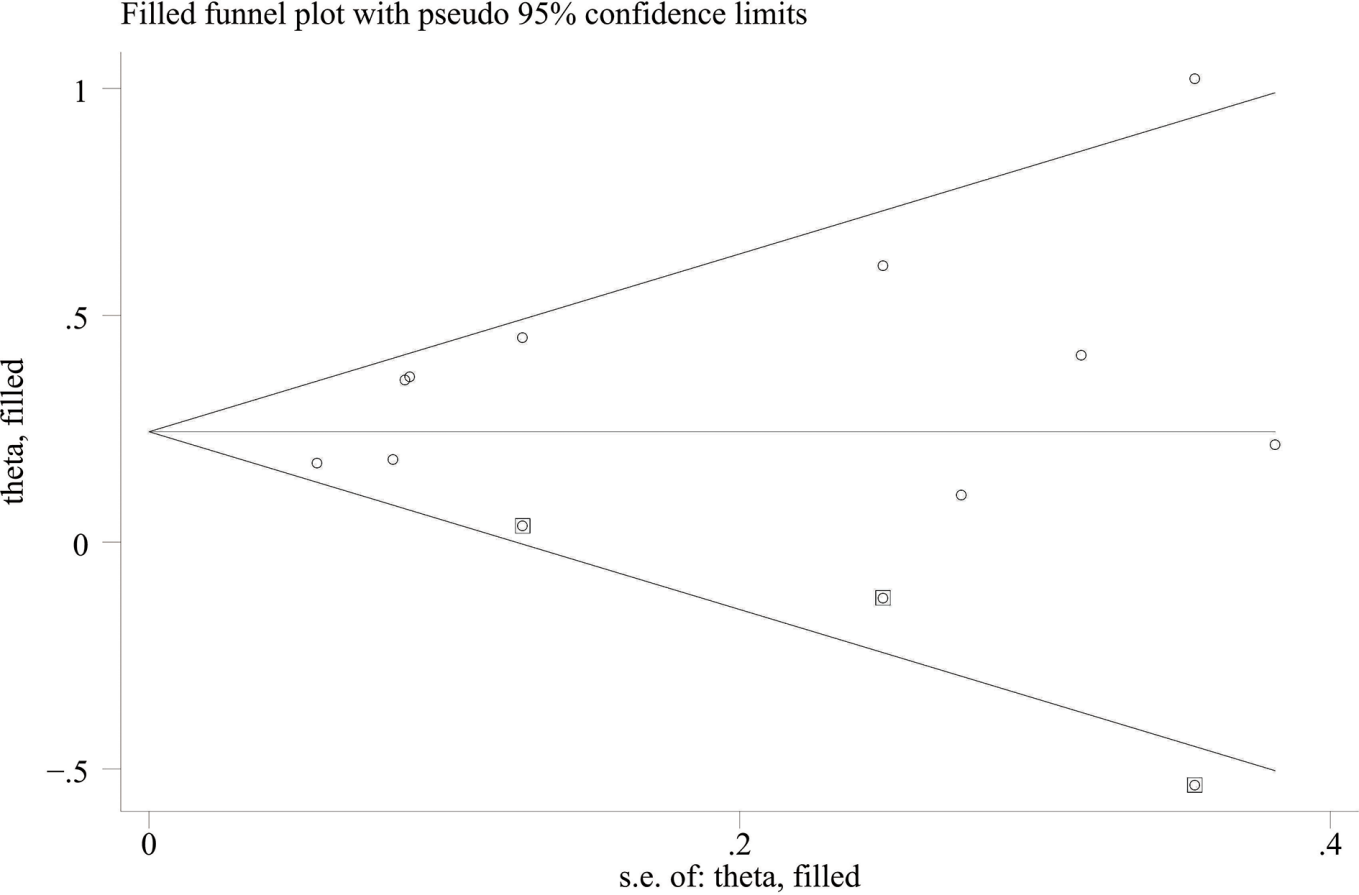
Trimming chart of univariate analysis outcomes of baseline CRP levels and PFS.

**Figure 7:**
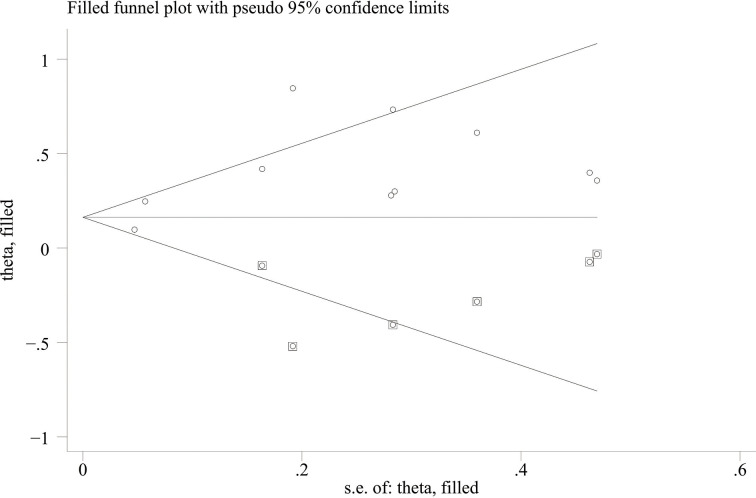
Trimming chart of multivariate analysis outcomes of baseline CRP levels and PFS.

When the high baseline CRP level group was compared with the low baseline CRP level group, the merged outcomes of univariate and multivariate analyses for worse PFS were 1.34 (95% CI = 1.15–1.55) and 1.34 (95% CI = 1.15–1.56), respectively (**Figure 5**). Sensitivity analysis revealed that both consequences of the study of Chasseuil et al. (35) contributed to a significant bias and, thus, must be discarded (**Supplementary Figures 9**-**12**). The funnel plot derived from the outcomes on PFS also showed plenty of missing negative results; thus, the trim-and-fill method was applied to rectify the pooled HR and 95% CI (**Supplementary Figures 13, 14**). The final corrected pooled outcomes for the PFS on univariate and multivariate analyses were 1.29 (95% CI = 1.15–1.45) and 1.20 (95% CI = 1.02–1.40), respectively (**Figures 6**, **7**). Further analysis indicated that 1 mg/dl was an appropriate cutoff value for baseline CRP to predict PFS in cancer patients receiving ICI treatment (univariate analysis: HR = 1.56, 95% CI = 1.24–1.97, *P* = 0.909; multivariate analysis: HR = 1.58, 95% CI = 1.23–2.03, *P* = 0.521) (**Supplementary Figure 15**).

### Subgroup Analysis

Further OS and PFS subgroup analyses were undertaken to appraise the correlation of CPR with cancer type, sample size, country, and ICIs (**Table 2**). Overall, the heterogeneity of the diverse subgroups dropped significantly compared with the whole group. Outcomes of the multivariate analysis component had more credibility, while the univariate analysis section was mainly employed for reference. As far as OS is concerned, high baseline CRP level was markedly correlated with worse OS in sample size <100 group (univariate analysis group: HR = 2.99, 95% CI = 2.90–4.71, *P* = 0.001; multivariate analysis group: HR = 4.13, 95% CI = 2.20–7.74, *P* = 0.006). The OS values of subgroups stratified by cancer type, country, or ICI type were not significantly different (1 < HR < 3 in the multivariate analysis group). No significant differences were observed in PFS for all subgroups (1 < HR < 2).

**Table 2:** Subgroup analyses.

	Subgroup	No. of cohorts	Univariate analysis HR (95% CI)	Heterogeneity test		No. of cohorts	Multivariate analysis HR (95% CI)	Heterogeneity test	
				I2	*P*			I2	*P*
OS									
Cancer type	NSCLC	6	1.62 (1.28, 2.05)	55.6	0.047				
	Melanoma	6	1.78 (1.54, 2.06)	0.0	0.510	9	1.84 (1.34, 2.52)	76.1	<0.001
	Others	9	1.78 (1.48, 2.14)	74.6	<0.001	11	2.00 (1.50, 2.66)	68.0	0.001
Sample size	>100	12	1.58 (1.43, 1.75)	57.2	0.007	12	1.57 (1.34, 1.83)	56.2	0.009
	<100	9	2.99 (1.90, 4.71)	69.9	0.001	8	4.13 (2.20, 7.74)	64.9	0.006
Country	Japan	10	2.92 (2.24, 3.80)	0.0	0.706	8	2.01 (1.29, 3.15)	61.8	0.011
	USA	4	1.70 (1.45, 2.00)	41.6	0.162	4	1.38 (1.17, 1.63)	0.0	0.611
	Others	7	1.49 (1.34, 1.65)	66.1	0.007	8	2.50 (1.71, 3.66)	82.6	<0.001
ICIs	Nivo	7	1.98 (1.42, 2.75)	70.9	0.002	7	1.49 (1.19, 1.87)	50.7	0.058
	ICIs	7	1.69 (1.37, 2.07)	60.6	0.019	4	1.64 (0.89, 3.02)	80.0	0.002
	Others	7	1.73 (1.47, 2.05)	64.7	0.009	9	2.54 (1.82, 3.55)	67.3	0.002
PFS									
Cancer type	NSCLC	6	1.39 (1.25, 1.54)	20.2	0.281	4	1.53 (1.22, 1.92)	0.0	0.649
	RCC	4	1.45 (1.02, 2.06)	50.5	0.109	4	1.30 (1.16, 1.44)	0.0	0.770
	Others					2	1.57 (0.75, 3.26)	93.0	0.000
Sample size	>100	6	1.32 (1.17, 1.49)	44.7	0.108	5	1.50 (1.19, 1.89)	57.9	0.050
	<100	4	1.52 (1.21, 1.91)	15.3	0.315	5	1.43 (1.03, 1.98)	45.5	0.119
Country	Japan	4	1.58 (1.32, 1.89)	0.0	0.416	8	1.36 (1.12, 1.65)	34.8	0.151
	Others	6	1.29 (1.17, 1.44)	47.0	0.129	2	1.68 (0.94, 3.01)	88.9	0.003
ICIs	Nivo	6	1.39 (1.13, 1.72)	47.7	0.089	6	1.34 (1.21, 1.48)	0.0	0.480
	ICIs	3	1.36 (1.14, 1.63)	49.4	0.138	2	1.11 (1.01, 1.21)	0.0	0.529
	Others	1	1.44 (1.21, 1.71)			2	1.85 (1.09, 3.13)	60.5	0.112

## Discussion

To our knowledge, this is the first meta-analysis investigating the correlation of baseline CRP level and outcomes of cancer patients treated with ICIs. The quality of the whole covered literature is generally favorable. As far as the pooled and rectified results are considered, baseline CRP level does correlate with OS and PFS in cancer patients managed with ICIs. The present studies also showed that patients with high levels of CRP or elevated CRP during treatment also had a worse OS and PFS compared with patients with low levels of CRP or decreased CRP (22, 28, 36, 46, 54, 55). Therefore, CRP is an excellent biomarker to predict the potency of ICI treatment, and more investigations are warranted to exploit the predictive value of CRP.

The clinical reference cutoff value for CRP is set as 0.5 mg/dl in some studies (40), but the cutoff value of high baseline CRP level remains controversial and various cutoff values have served in the literature. Our study permitted different cutoff values and the most popular applied cutoff value is still 1.0 mg/dl (21, 24, 29, 34, 35, 42, 43, 47, 49–51). The principal cause of the discrepancy in cutoff values is that some studies employed the median CRP level as the cutoff value, just to equalize the number of individuals in the high CRP and low CRP groups. If it is applied to the clinic, a uniform cutoff is mandatory, and 1.0 mg/dl deserves to be under consideration. We have conducted a further analysis about the median CRP level of cancer patients in all studies, and available data revealed that the majority of the patients have median CRP level <1 mg/dl. Therefore, 1 mg/dl may be a popular and suitable cutoff value for CRP. Of course, the CRP level may differ in different types of cancer, so more high–quality prospective clinical studies are warranted to determine the most suitable cutoff value of baseline CRP for different cancers.

The reason why CRP levels are closely associated with the prognosis of cancer patients receiving ICI treatment remains unclear. It may be attributed essentially to several mechanisms according to a comprehensive review of the related literature and clinical experience. It has previously been observed that CRP can directly suppress T cell (56) and dendritic cell (57), thereby influencing the effect of ICIs by impairing the intrinsic and acquired immunity of cancer patients. At the same time, CRP can promote inflammatory response (58), which will suppress immune function, and facilitate cancer multiplication and transmigration (59). CRP can also foster the formation of the tumor microenvironment (TME) (60, 61), which might impair the effect of ICIs. What is more, CRP is an indicator of body damage (62). It means that, compared with patients with low pretreatment CRP level, patients with high pretreatment CRP level perhaps have worse health and cancer circumstances, which will also affect the effectiveness of ICIs.

Subgroup analysis will inevitably decrease the number of studies, and it is more plausible that the joint outcomes would be biased. Given the magnitude of the *P*–value ascending, it seems that OS–related heterogeneity is strongly affected by potentially country–specific differences, which may be ethnic differences and concrete lifestyles. PFS–related heterogeneity is significantly influenced by cancer type and ICI category. It might be because of the various resistance mechanisms, proliferation, and migration rates of different cancers, and varying efficacy of specific ICIs.

Overall, CRP is an independent and desirable predictive biomarker of OS and PFS in cancer patients receiving ICI treatment, and quantifying CRP through blood examination is convenient and less invasive. The combination of CRP and other predictors to form a predictive model is also a feasible idea that merits investigation (53, 63, 64). In addition, for cancer patients with high CRP levels, the handling of inflammation and TME should be of particular attention to reduce resistance to ICIs (17). Inflammation can be addressed by causal therapy, and TME might be addressed by TME modulation that is being investigated (65). As confirmed by clinical literature, statin therapy can diminish CRP levels (66). Therefore, statin therapy might be also applied to high CRP cancer patients to improve ICI treatment. What is more, a recent study revealed that moderation–vigorous physical activity (MVPA) could reduce CRP level in breast cancer patients (67). Whether MVPA may be recommended to cancer patients with high CRP level before immunotherapy deserves further studies.

There are some limitations to be considered. First, some researchers do not publish adverse outcomes as they consider them meaningless, leading to a few unavoidable publication biases. Hence, we proceeded with the trim–and–fill method to acquire adjusted results to clarify this association. Secondly, because we only included studies published in English, we may miss some relevant studies. The present application with ICIs is predominately administered in limited types of cancers (68). The value of CRP in predicting the prognosis of breast cancer (69), prostate cancer (14), pancreatic cancer (70), colorectal cancer (71), and other cancers has been reported. However, these studies did not focus on the efficacy of ICIs. What is more, most of the selected papers were retrospective and from Japan. More prospective studies are needed to assess the predictive value of CRP for different cancer patients in different countries.

## Conclusion

In summary, regardless of cancer type, high baseline CRP level is significantly correlated to worse OS and PFS in cancer patients treated with ICIs. Our study indicates that the baseline CRP level is a useful predictor and 1 mg/dl may be a suitable cutoff value to identify cancer patients who may benefit from ICI treatment, thereby helping to lead decisions in personalized treatments.

## Data Availability Statement

The original contributions presented in the study are included in the article/**Supplementary Material**. Further inquiries can be directed to the corresponding author.

## Author Contributions

All authors had full access to the data in the study and took responsibility for the integrity and authenticity of the data. C–LH formulated the study objective, conceptualized the study, performed the statistical analysis, and interpreted the results. G–XM and Z–ND designed the protocol of the systematic reviews. Z–RD, Z–QC, and J–GH provided essential guidance to the protocols and modified them. C–LH, L–JY, and HL, respectively, evaluated the availability of each study and disagreements were discussed and addressed with B–WT. L–SY and J–SX performed the methodology, data collection, and data validation. C–LH and TL guided the task of formal statistical analysis and analysis of the data. C–LH and TL contributed to outlining the manuscript and drafting the manuscript. C–LH and TL verified the underlying data. TL supervised and coordinated the study. All authors have read and approved the final version.

## Funding

This work was supported by the Taishan Scholars Program for Young Expert of Shandong Province (tsqn20161064), the National Natural Science Foundation of China (81874178 and 82073200), Major Basic Research of Shandong Provincial Natural Science Foundation (Grant No. ZR202105070027), and Founds for Independent Cultivation of Innovative Team from Universities in Jinan (Grant No. 2020GXRC023).

## Conflict of Interest

The authors declare that the research was conducted in the absence of any commercial or financial relationships that could be construed as a potential conflict of interest.

## Publisher’s Note

All claims expressed in this article are solely those of the authors and do not necessarily represent those of their affiliated organizations, or those of the publisher, the editors and the reviewers. Any product that may be evaluated in this article, or claim that may be made by its manufacturer, is not guaranteed or endorsed by the publisher.

## Abbreviations

CRP, C–reactive protein; ICIs, immune checkpoint inhibitors; PFS, progression–free survival; OS, overall survival; PD–1, programmed cell death 1; PD–L1, programmed cell death ligand 1; CTLA–4, cytotoxic T lymphocyte–associated protein 4; PRISMA, Preferred Reporting Items for Systematic Reviews and Meta–Analyses; NOS, Newcastle–Ottawa Scale; HR, hazard ratio; 95% CI, 95% confidence interval; GPS, Glasgow prognostic score; Oct, October; No., number; NR, not reported; UC, urothelial cancer; ESCC, esophageal squamous cell carcinoma; HNSCC, head and neck squamous cell carcinoma; NSCLC, non–small cell lung cancer, RCC, renal cell carcinoma; GC, gastric cancer; SGC, salivary gland carcinoma; HCC, hepatocellular carcinoma; Pembro, pembrolizumab; Nivo, nivolumab; Ipi, ipilimumab; Camre, camrelizumab; Durva, durvalumab; Atezo, atezolizumab; TME, tumor microenvironment; MVPA, moderation–vigorous physical activity.
