# Supplementary figures and images for: The Predictive Potential of the Baseline C-Reactive Protein Levels for the Efficiency of Immune Checkpoint Inhibitors in Cancer Patients: A Systematic Review and Meta-Analysis
Source: Front Immunol. 2022 Feb 8;13:827788. doi: 10.3389/fimmu.2022.827788 (PMC8861087; doi:10.3389/fimmu.2022.827788)

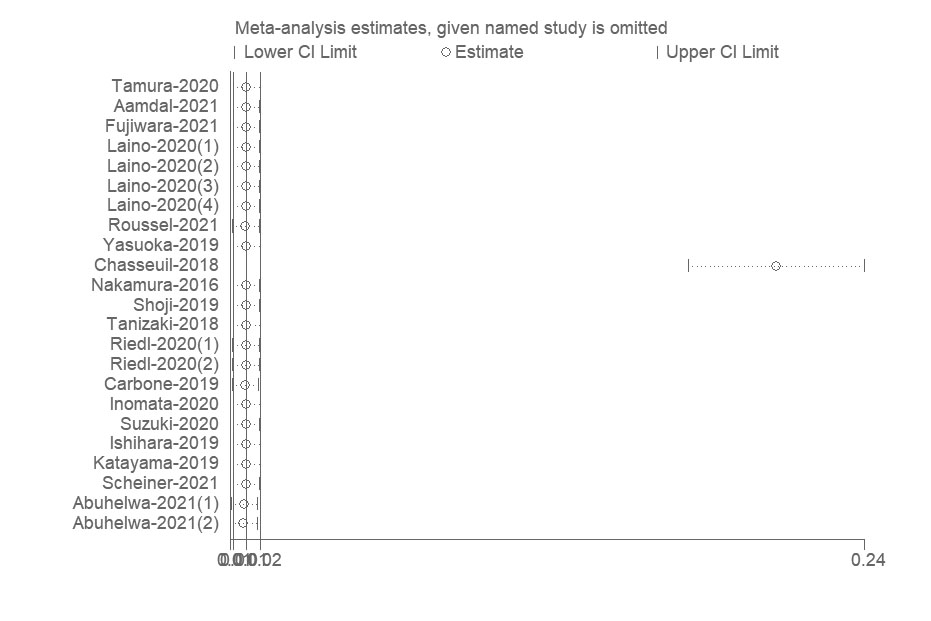

Supplement: Supplementary file 1 [file Image_1.jpeg]

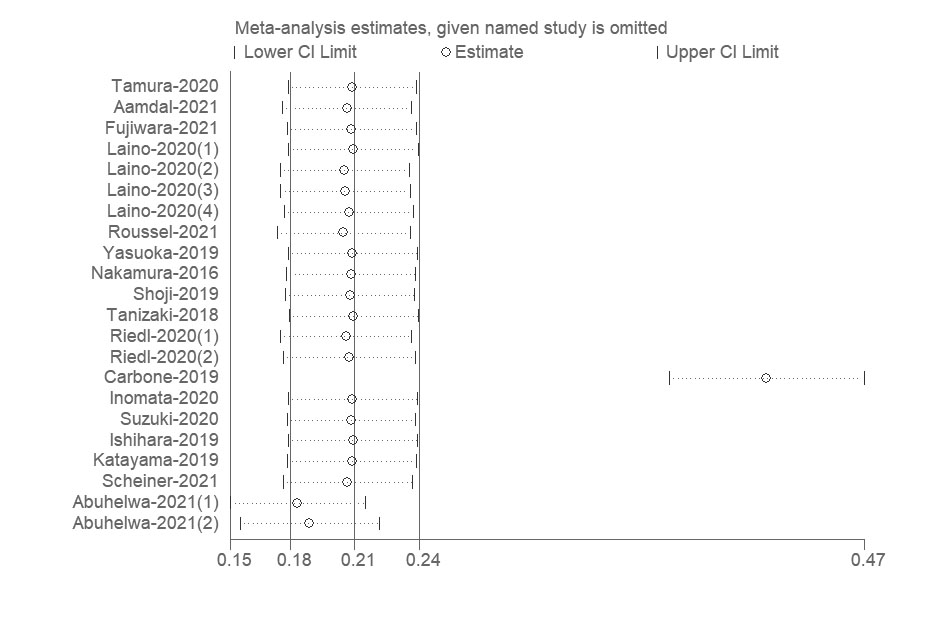

Supplement: Supplementary file 2 [file Image_2.jpeg]

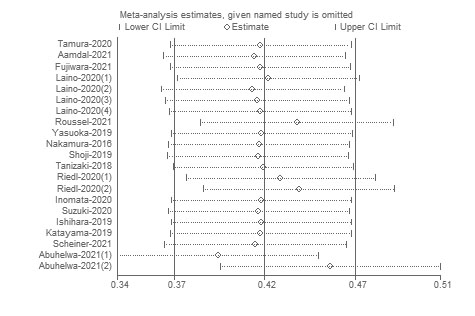

Supplement: Supplementary file 3 [file Image_3.jpeg]

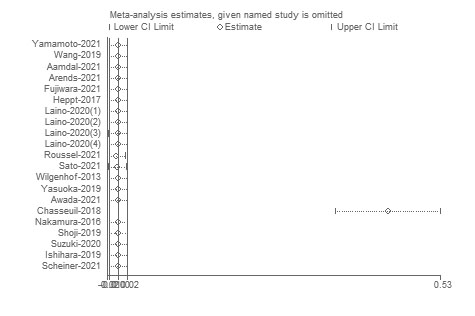

Supplement: Supplementary file 4 [file Image_4.jpeg]

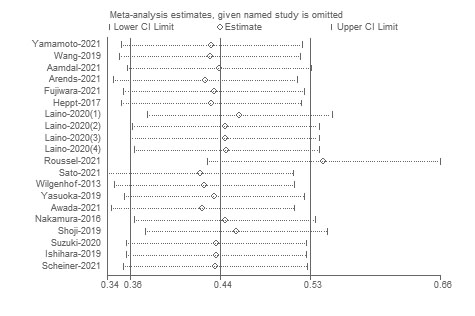

Supplement: Supplementary file 5 [file Image_5.jpeg]

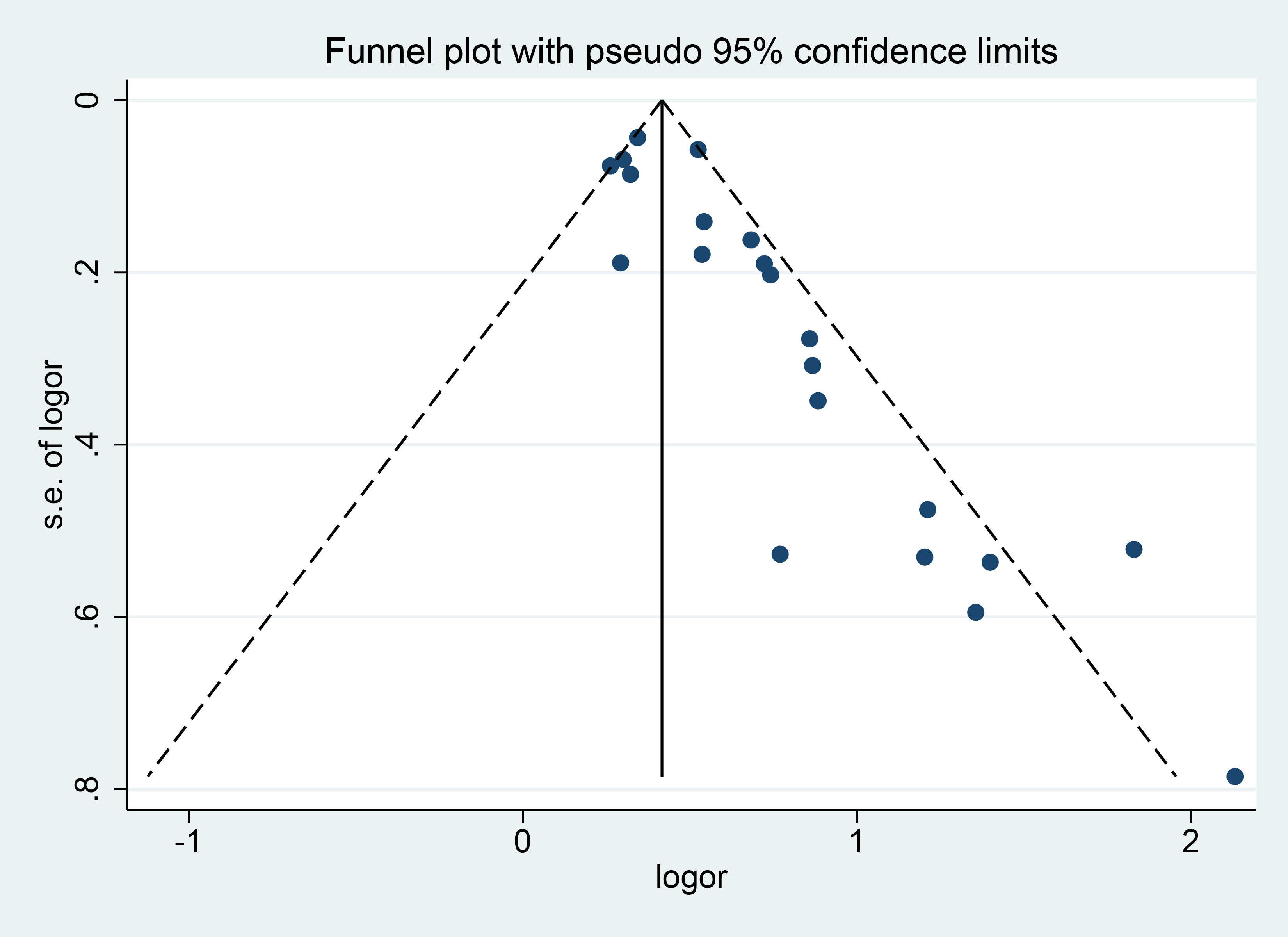

Supplement: Supplementary file 6 [file Image_6.jpeg]

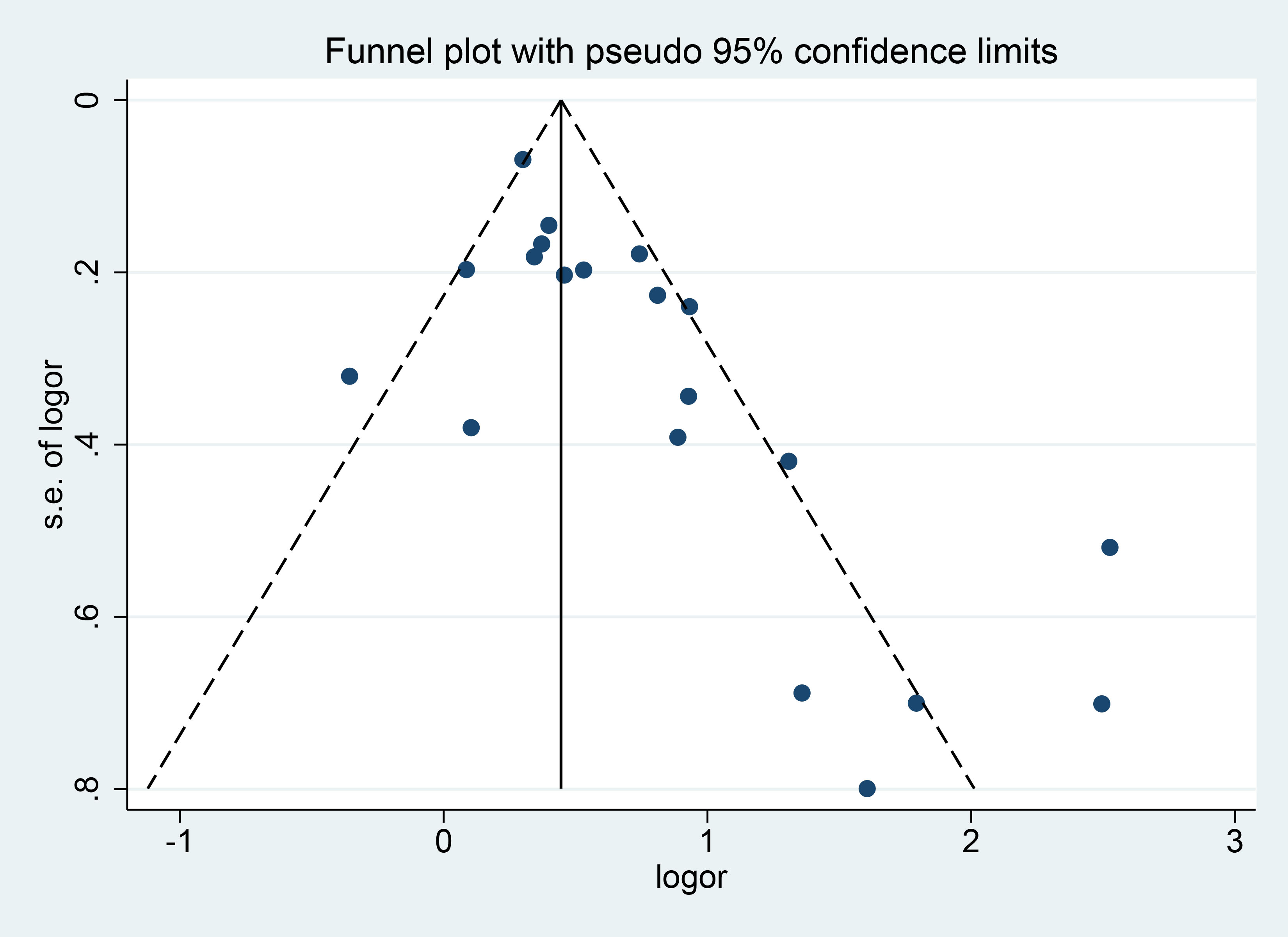

Supplement: Supplementary file 7 [file Image_7.jpeg]

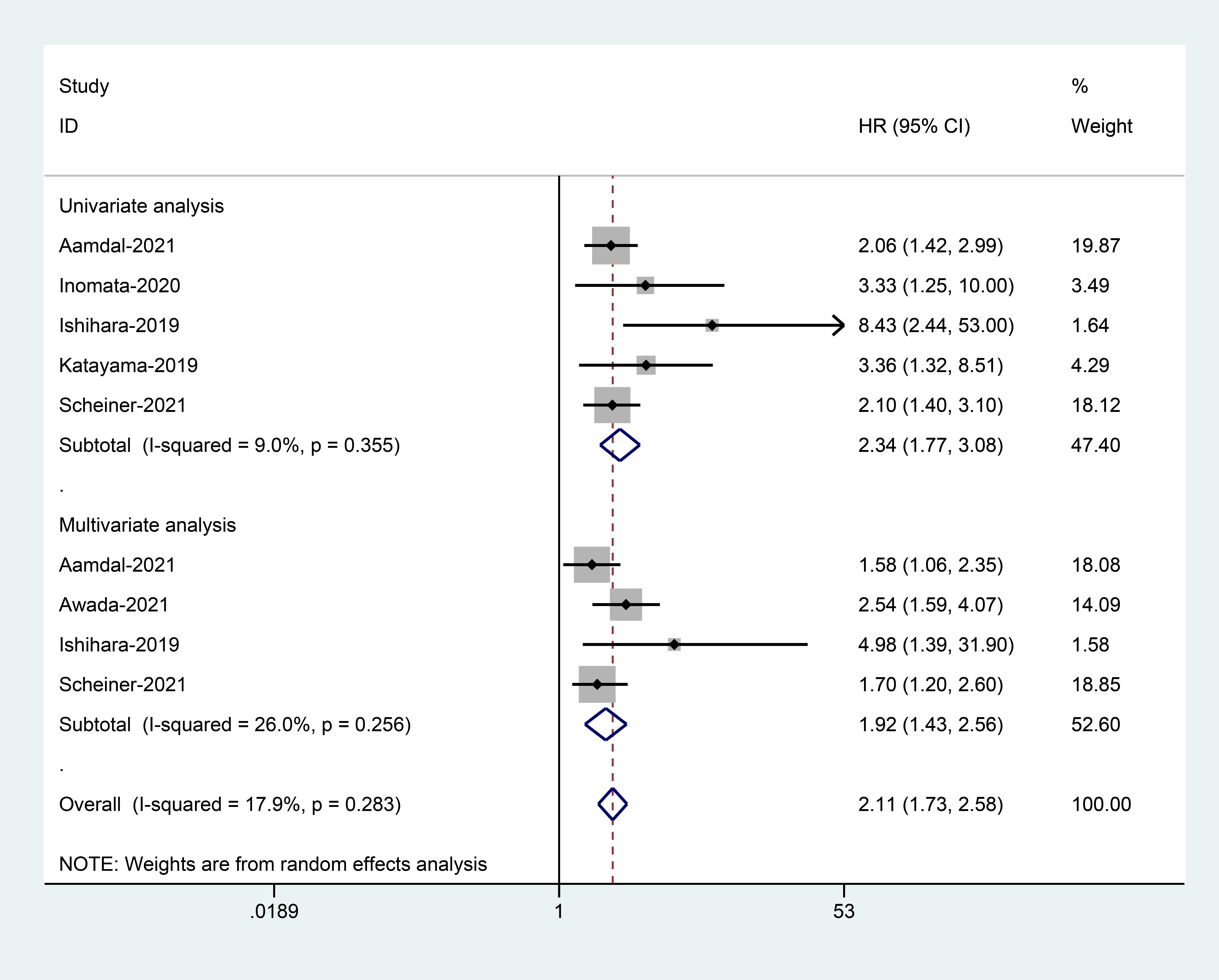

Supplement: Supplementary file 8 [file Image_8.jpeg]

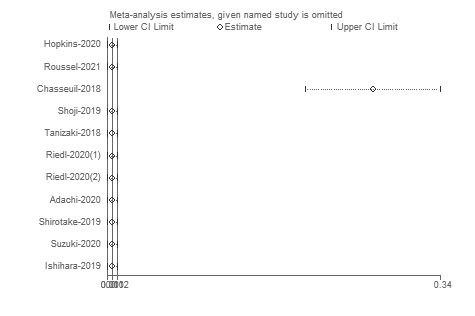

Supplement: Supplementary file 9 [file Image_9.png]

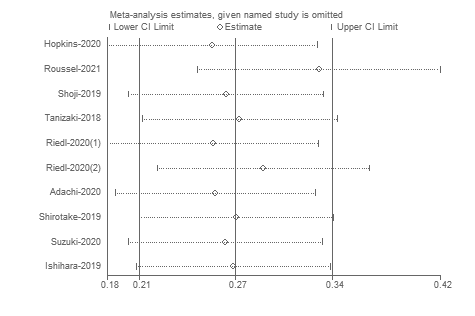

Supplement: Supplementary file 10 [file Image_10.png]

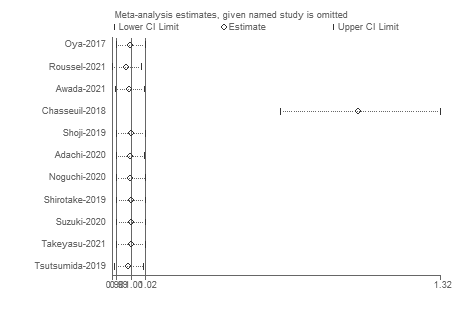

Supplement: Supplementary file 11 [file Image_11.png]

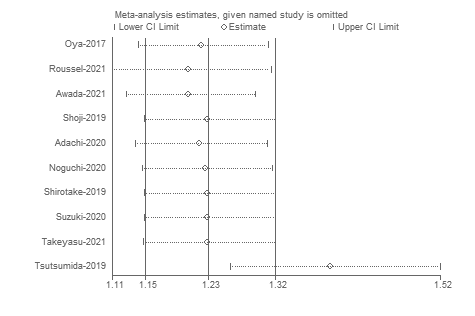

Supplement: Supplementary file 12 [file Image_12.png]

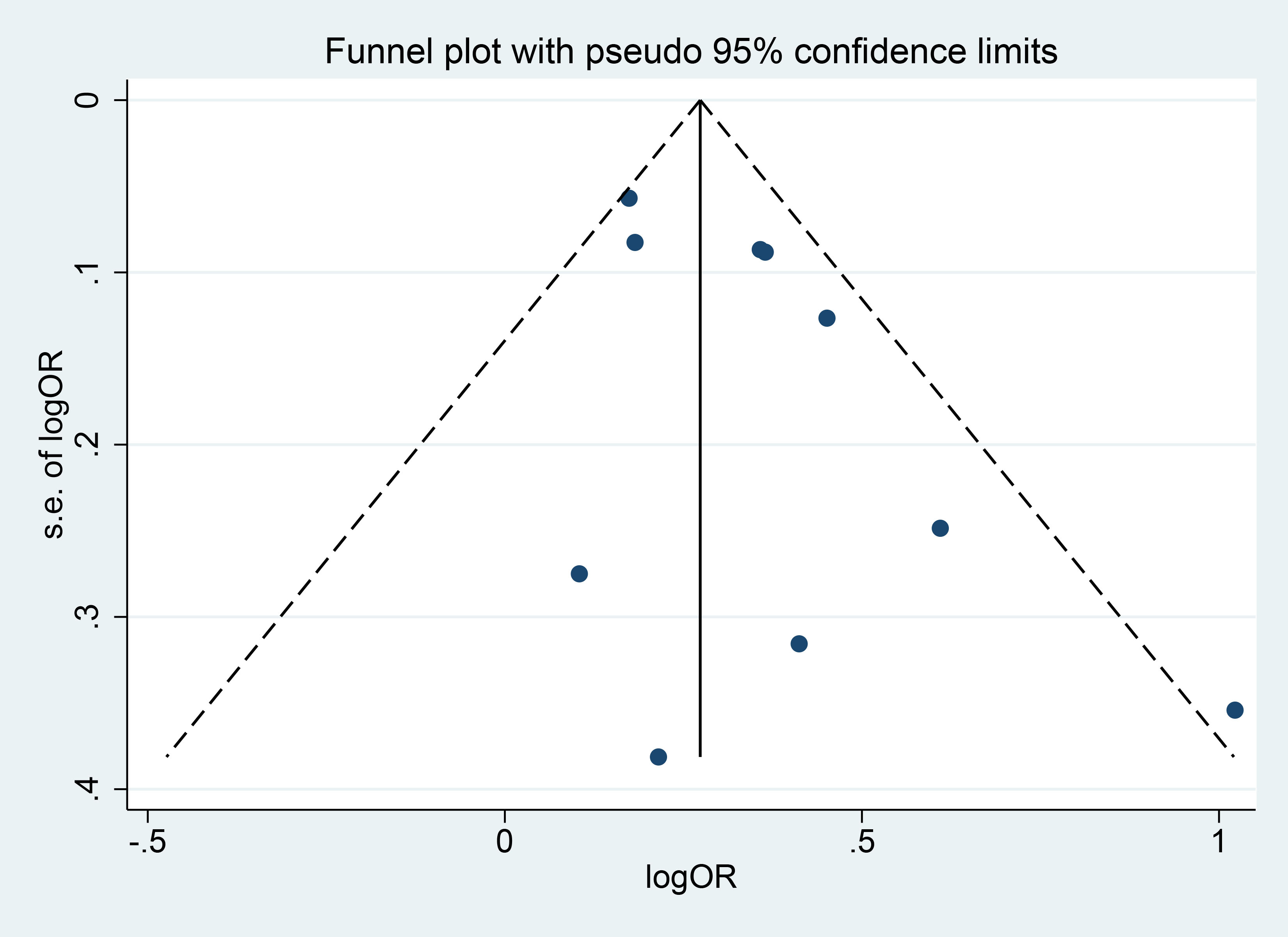

Supplement: Supplementary file 13 [file Image_13.jpeg]

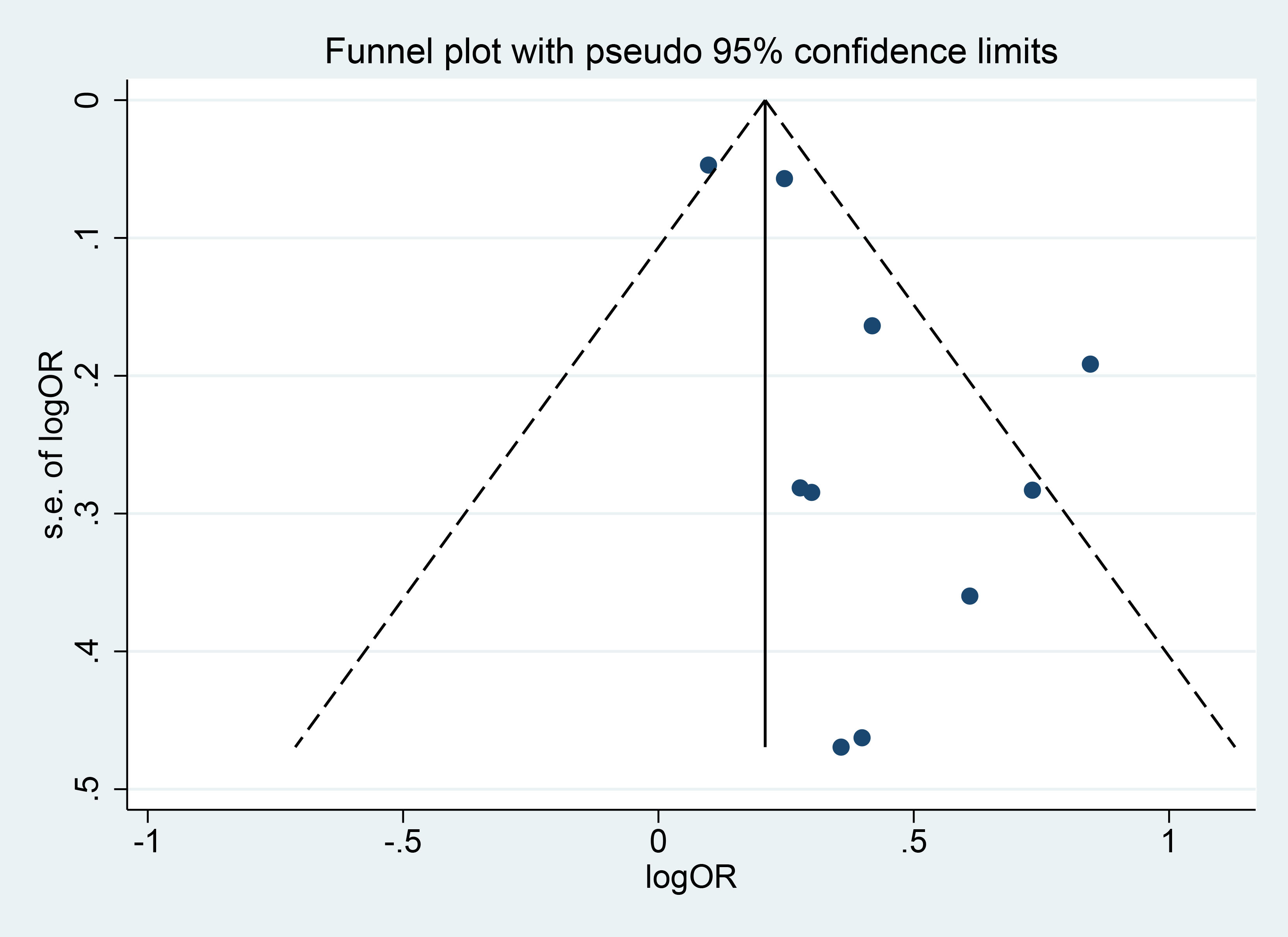

Supplement: Supplementary file 14 [file Image_14.jpeg]

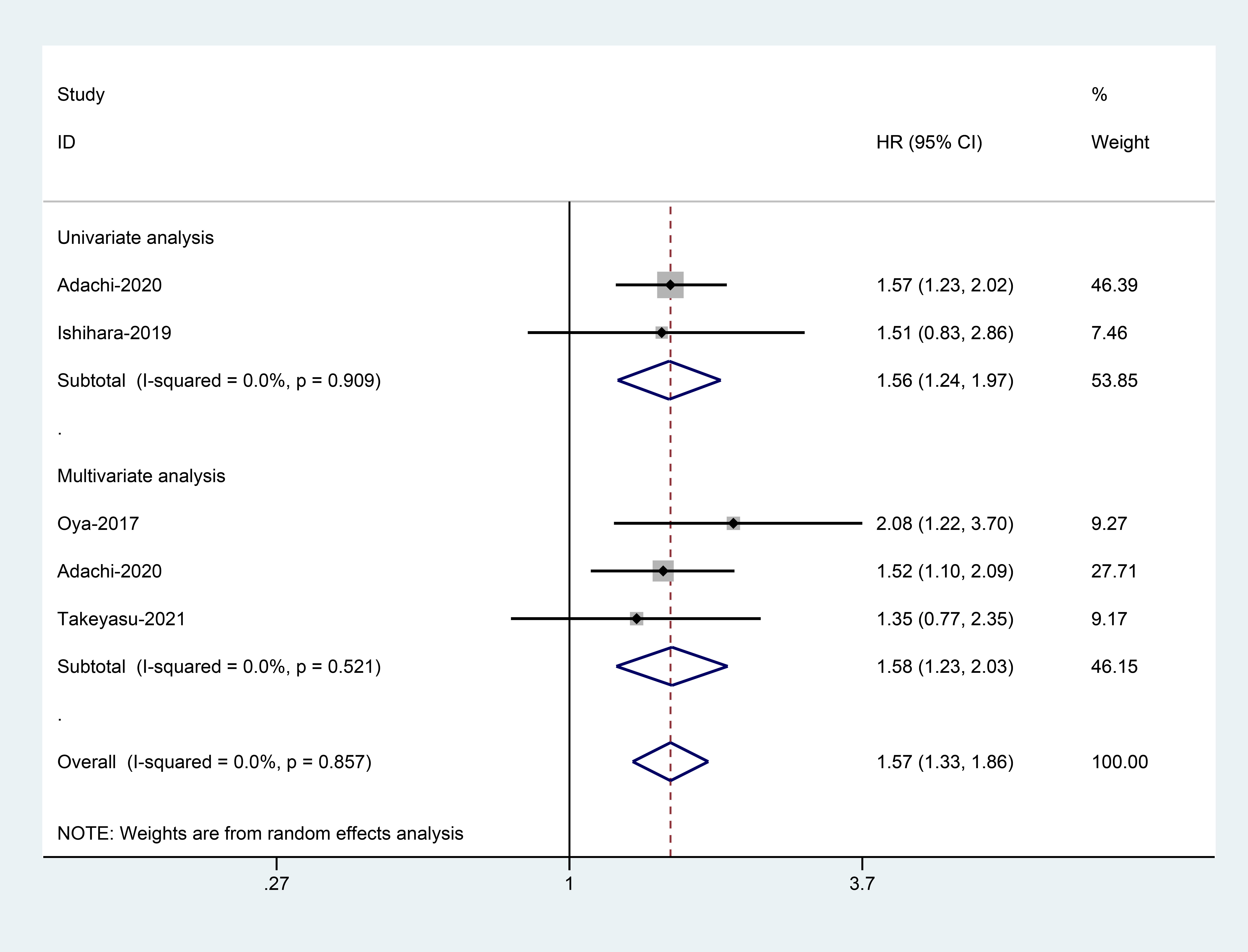

Supplement: Supplementary file 15 [file Image_15.jpeg]
