# Supplementary material for: The Predictive Potential of the Baseline C-Reactive Protein Levels for the Efficiency of Immune Checkpoint Inhibitors in Cancer Patients: A Systematic Review and Meta-Analysis
Source: Front Immunol. 2022 Feb 8;13:827788. doi: 10.3389/fimmu.2022.827788 (PMC8861087; doi:10.3389/fimmu.2022.827788)
Supplement: Supplementary file 16 [file Table_1.docx]

**Supplementary Table 1: Database-specific search strategy for Embase**

| P (population) | Keywords searched for in abstract and title | Carcinoma OR neoplasm OR malignancy OR cancer |
| --- | --- | --- |
|  | Emtree | none |
| I (intervention) | Keywords searched for in abstract and title | immune checkpoint inhibitor OR ICIs OR avelumab OR durvalumab OR tremelimumab OR pembrolizumab OR camrelizumab OR ipilimumab OR tislelizumab OR SHR-1210 OR toripalimab OR penpulimab OR nivolumab OR atezolizumab OR PD-1 OR PD-L1 OR CTLA-4 |
|  | Emtree | none |
| C (comparison) | Keywords searched for in abstract, title | C-reactive protein OR CRP |
|  | Emtree | none |
| O (outcome) | none | |
| S (study design) | none | |
| Additional limits | Limits to English language only | |

**Supplementary Retrieval Methods**

**In EMBASE**

('immune checkpoint inhibitor':ti,ab,kw OR atezolizumab:ti,ab,kw OR 'cytotoxic t lymphocyte antigen 4':ti,ab,kw OR 'pd 1':ti,ab,kw OR 'pd l1':ti,ab,kw OR 'shr 1210':ti,ab,kw OR toripalimab:ti,ab,kw OR nivolumab:ti,ab,kw OR penpulimab:ti,ab,kw OR icis:ti,ab,kw OR avelumab:ti,ab,kw OR camrelizumab:ti,ab,kw OR tislelizumab:ti,ab,kw OR ipilimumab:ti,ab,kw OR durvalumab:ti,ab,kw OR pembrolizumab:ti,ab,kw OR ticilimumab:ti,ab,kw) AND (cancer:ti,ab,kw OR neoplasm:ti,ab,kw OR malignancy:ti,ab,kw OR carcinoma:ti,ab,kw) AND ('c reactive protein':ti,ab,kw OR crp:ti,ab,kw)

Limits: Removing potential duplicates through Endnote X9.

**In Pubmed**

((((Carcinoma[Title/Abstract]) OR (neoplasm[Title/Abstract])) OR (malignancy[Title/Abstract])) OR (cancer[Title/Abstract])) AND (((((((((((((((((immune checkpoint inhibitor[Title/Abstract]) OR (CTLA-4[Title/Abstract])) OR (PD-L1[Title/Abstract])) OR (PD-1[Title/Abstract])) OR (atezolizumab[Title/Abstract])) OR (nivolumab[Title/Abstract])) OR (penpulimab[Title/Abstract])) OR (toripalimab[Title/Abstract])) OR (SHR-1210[Title/Abstract])) OR (tislelizumab[Title/Abstract])) OR (ipilimumab[Title/Abstract])) OR (camrelizumab[Title/Abstract])) OR (pembrolizumab[Title/Abstract])) OR (tremelimumab[Title/Abstract])) OR (durvalumab[Title/Abstract])) OR (avelumab[Title/Abstract])) OR (ICIs[Title/Abstract])) AND （(C-reactive protein[Title/Abstract]) OR (CRP [Title/Abstract])）

Limits: Removing potential duplicates through Endnote X9.

**In Cochrane Library literature**

"carcinoma" OR "neoplasma" OR malignancy OR "Cancer" in Title Abstract Keyword AND immune checkpoint inhibitor OR ICIs OR avelumab OR durvalumab OR tremelimumab OR pembrolizumab OR camrelizumab OR ipilimumab OR tislelizumab OR SHR-1210 OR toripalimab OR penpulimab OR nivolumab OR atezolizumab OR PD-1 OR PD-L1 OR CTLA-4 in Title Abstract Keyword AND "C-reactive protein" OR "CRP" in Title Abstract Keyword

Limits: Removing potential duplicates through Endnote X9.
