# Supplementary material for: The Predictive Potential of the Baseline C-Reactive Protein Levels for the Efficiency of Immune Checkpoint Inhibitors in Cancer Patients: A Systematic Review and Meta-Analysis
Source: Front Immunol. 2022 Feb 8;13:827788. doi: 10.3389/fimmu.2022.827788 (PMC8861087; doi:10.3389/fimmu.2022.827788)
Supplement: Supplementary file 17 [file Table_2.docx]

**Supplementary Table 2: Details of Newcastle-Ottawa Scale**

|  |  | **Selection** | **Comparability** | **Exposure** |  |
| --- | --- | --- | --- | --- | --- |
| Yamamoto-2021 |  | ☆☆☆ | ☆☆ | ☆☆ | 7 |
| Tamura-2020 |  | ☆☆☆ |  | ☆☆ | 5 |
| Wang-2019 |  | ☆☆ | ☆☆ | ☆☆ | 6 |
| Aamdal-2021 |  | ☆☆ | ☆☆ | ☆☆ | 6 |
| Arends-2021 |  | ☆☆ | ☆☆ | ☆☆ | 6 |
| Fujiwara-2021 |  | ☆☆☆ | ☆☆ | ☆☆ | 7 |
| Heppt-2017 |  | ☆☆☆ | ☆☆ | ☆☆ | 7 |
| Hopkins-2020 |  | ☆☆ | ☆☆ | ☆☆ | 6 |
| Laino-2020 |  | ☆☆ | ☆☆ | ☆☆ | 6 |
| Oya-2017 |  | ☆ | ☆☆ | ☆☆ | 5 |
| Roussel-2021 |  | ☆☆☆ | ☆☆ | ☆☆ | 7 |
| Sato-2021 |  | ☆☆ |  | ☆☆ | 4 |
| Wilgenhof-2013 |  | ☆☆☆ |  | ☆☆ | 5 |
| Yasuoka-2019 |  | ☆☆☆ |  | ☆☆ | 5 |
| Awada-2021 |  | ☆☆ | ☆☆ | ☆☆ | 6 |
| Chasseuil-2018 |  | ☆☆ | ☆ | ☆☆ | 5 |
| Nakamura-2016 |  | ☆☆☆ | ☆☆ | ☆☆ | 7 |
| Niwa-2020 |  | ☆☆☆ | ☆☆ | ☆☆ | 7 |
| Shoji-2019 |  | ☆☆☆ | ☆☆ | ☆☆ | 7 |
| Tanizaki-2018 |  | ☆☆☆ | ☆☆ | ☆☆ | 7 |
| Riedl-2020 |  | ☆☆☆ | ☆☆ | ☆☆ | 7 |
| Carbone-2019 |  | ☆☆ |  | ☆☆ | 4 |
| Adachi-2020 |  | ☆☆ | ☆☆ | ☆☆ | 6 |
| Inomata-2020 |  | ☆☆☆ |  | ☆☆ | 5 |
| Noguchi-2020 |  | ☆☆☆ | ☆☆ | ☆☆ | 7 |
| Shirotake-2019 |  | ☆☆☆ | ☆☆ | ☆☆ | 7 |
| Suzuki-2020 |  | ☆☆☆ | ☆☆ | ☆☆ | 7 |
| Takeyasu-2021 |  | ☆☆ | ☆☆ | ☆☆ | 6 |
| Tsutsumida-2019 |  | ☆ | ☆☆ | ☆☆ | 5 |
| Ishihara-2019 |  | ☆☆ | ☆☆ | ☆☆ | 6 |
| Katayama-2019 |  | ☆☆☆ |  | ☆☆ | 5 |
| Scheiner-2021 |  | ☆☆☆ | ☆☆ | ☆☆ | 7 |
| Abuhelwa-2021 |  | ☆☆ | ☆☆ | ☆☆ | 6 |
