# Supplementary material for: The Predictive Potential of the Baseline C-Reactive Protein Levels for the Efficiency of Immune Checkpoint Inhibitors in Cancer Patients: A Systematic Review and Meta-Analysis
Source: Front Immunol. 2022 Feb 8;13:827788. doi: 10.3389/fimmu.2022.827788 (PMC8861087; doi:10.3389/fimmu.2022.827788)
Supplement: Supplementary file 18 [file Table_3.docx]

Supplementary Table 3: Cut-off value of all included studies

| Study | Cut-off value |
| --- | --- |
| Yamamoto-2021 | 1.0 mg/dl |
| Tamura-2020 | Median CRP level: 1.06 mg/dl |
| Wang-2019 | 0.5 mg/dl |
| Aamdal-2021 | 1.0 mg/dl |
| Arends-2021 | Median CRP level ^(a)^ |
| Fujiwara-2021 | 0.5 mg/dl |
| Heppt-2017 | 0.5 mg/dl |
| Hopkins-2020 | 3 mg/dl |
| Laino-2020 | Median CRP level ^(b)^ |
| Oya-2017 | 1.0 mg/dl |
| Roussel-2021 | Continuous analysis: per 2.5 mg/dl^(c)^ |
| Sato-2021 | 0.5 mg/dl |
| Wilgenhof-2013 | 2.5 mg/dl |
| Yasuoka-2019 | 0.5 mg/dl |
| Awada-2021 | 1.0 mg/dl |
| Chasseuil-2018 | 1.0 mg/dl |
| Nakamura-2016 | 0.29 mg/dl |
| Niwa-2020 | NR ^(d)^ |
| Shoji-2019 | 0.51 mg/dl |
| Tanizaki-2018 | 0.41 mg/dl |
| Riedl-2020 | Continuous analysis: per doubling |
| Carbone-2019 | NR |
| Adachi-2020 | 1.0 mg/dl |
| Inomata-2020 | 1.0 mg/dl |
| Noguchi-2020 | 1.5 mg/dl |
| Shirotake-2019 | Median CRP level: 0.67 mg/dl |
| Suzuki-2020 | 2.1 mg/dl |
| Takeyasu-2021 | 1.0 mg/dl |
| Tsutsumida-2019 | Continuous analysis: per 1.0 mg/L |
| Ishihara-2019 | 1.0 mg/dl |
| Katayama-2019 | 1.0 mg/dl |
| Scheiner-2021 | 1.0 mg/dl |
| Abuhelwa-2021 | NR |

NR: Not Report.

1. Only mentioned patients stratified according to the median baseline CRP levels, but didn’t give the value of median baseline CRP levels.
2. Patients in Checkmate-066: 0.53 mg/dl, Patients in Checkmate-067: 0.575 mg/dl
3. Per means it used the continuous analysis
4. Didn’t report the unit of CRP.
