# Supplementary material for: The Predictive Potential of the Baseline C-Reactive Protein Levels for the Efficiency of Immune Checkpoint Inhibitors in Cancer Patients: A Systematic Review and Meta-Analysis
Source: Front Immunol. 2022 Feb 8;13:827788. doi: 10.3389/fimmu.2022.827788 (PMC8861087; doi:10.3389/fimmu.2022.827788)
Supplement: Supplementary file 19 [file Table_4.docx]

**Supplementary Table 4: Median CRP of included studies**

| Median CRP | Reference number | Number of studies | Number of patients |
| --- | --- | --- | --- |
| < 1mg/dl | 24,26,29,30,35.36,37,43,46,49 | 10 | 2501 |
| > 1mg/dl | 22,41,44,45,47,50,52,53 | 8 | 1541 |
