# Supplementary material for: The Predictive Potential of the Baseline C-Reactive Protein Levels for the Efficiency of Immune Checkpoint Inhibitors in Cancer Patients: A Systematic Review and Meta-Analysis
Source: Front Immunol. 2022 Feb 8;13:827788. doi: 10.3389/fimmu.2022.827788 (PMC8861087; doi:10.3389/fimmu.2022.827788)
Supplement: Supplementary file 21 [file Table_6.docx]

**Supplementary material Legends**

Supplementary Table 1: Database-specific search strategy for Embase

Supplementary Table 2: Details of Newcastle-Ottawa Scale

Supplementary Table 3: Cut-off value of all included studies

Supplementary Table 4: Median CRP of included studies

Supplementary PRISMA Checklist

Supplementary Figure 1: Sensitivity analysis outcomes of univariate analysis outcomes of baseline CRP levels and OS before drop outcome of Chasseuil-2018.

Supplementary Figure 2: Sensitivity analysis outcomes of univariate analysis outcomes of baseline CRP levels and OS after drop outcome of Chasseuil-2018.

Supplementary Figure 3: Sensitivity analysis outcomes of univariate analysis outcomes of baseline CRP levels and OS after drop outcomes of Chasseuil-2018 and Carbone-2019.

Supplementary Figure 4: Sensitivity analysis outcomes of multivariate analysis outcomes of baseline CRP levels and OS before drop outcome of Chasseuil-2018.

Supplementary Figure 5: Sensitivity analysis outcomes of multivariate analysis outcomes of baseline CRP levels and OS after drop outcome of Chasseuil-2018.

Supplementary Figure 6: Funnel diagram obtained from univariate analysis outcomes of baseline CRP levels and OS (Point= Existing outcomes)

Supplementary Figure 7: Funnel diagram obtained from multivariate analysis outcomes of baseline CRP levels and OS

Supplementary Figure 8: Forest plot of HR and 95% CI concerning the relationship between baseline CRP levels and OS for all studies with 1 mg/dl cut-off value

Supplementary Figure 9: Sensitivity analysis outcomes of univariate analysis outcomes of baseline CRP levels and PFS before drop outcome of Chasseuil-2018.

Supplementary Figure 10: Sensitivity analysis outcomes of univariate analysis outcomes of baseline CRP levels and PFS after drop outcome of Chasseuil-2018.

Supplementary Figure 11: Sensitivity analysis outcomes of multivariate analysis outcomes of baseline CRP levels and PFS before drop outcome of Chasseuil-2018.

Supplementary Figure 12: Sensitivity analysis outcomes of multivariate analysis outcomes of baseline CRP levels and PFS after drop outcome of Chasseuil-2018.

Supplementary Figure 13: Funnel diagram obtained from univariate analysis outcomes of baseline CRP levels and PFS

Supplementary Figure 14: Funnel diagram obtained from multivariate analysis outcomes of baseline CRP levels and PFS

Supplementary Figure 15: Forest plot of HR and 95% CI concerning the relationship between baseline CRP levels and PFS for all studies with 1 mg/dl cut-off value.
